# Beilstein Journal of Nanotechnology

**DOI:** 10.3762/bjnano.1.1

**Published:** 2010-11-22

**Authors:** Thomas Schimmel

**Affiliations:** 1 Karlsruhe Institute of Technology (KIT), Institute of Applied Physics, 76128 Karlsruhe, Germany

Nanotechnology is considered one of the key technologies of the 21^st^ century. Increasingly smaller structures are gaining technological and economic importance. The scope of applications ranges from high-performance materials and coatings to car paints and catalysis, from bionic surfaces to medical applications, from nano-electronics to data storage devices, to mention only a few examples.

Rapid progress in nanotechnology is driven by two major factors. On the one hand, scientific progress allows the development of completely new products, processes and technologies. On the other hand, further progress in existing key technologies increasingly depends on the understanding and control of functional structures on the nanometer scale. Frequently, technological progress is hampered by a lack of understanding of structures on this length scale. To be successful, nanotechnology has to rely on a close cooperation of a number of different disciplines – including physics, chemistry and biology as well as engineering sciences and materials research.

At the same time, the nanosciences are a vivid and rapidly developing area of research, continuously finding and understanding new phenomena and effects related to the small length scale. Things do not only become smaller, they behave differently, opening fascinating perspectives for new science and novel applications and technologies. Nano-photonics, quantum electronics, the lotus effect and the fascinating properties of graphene are only a few well-known examples.

The Beilstein Journal of Nanotechnology provides a platform for the effective exchange and dissemination of cutting-edge results in the broad area of nanosciences and nanotechnology, including nanoscale physics, chemistry and biology, as well as engineering sciences, surface sciences, materials research and semiconductor technologies. It includes synthetic approaches and experimental work as well as computational nanoscience and theoretical aspects.

The Beilstein Journal of Nanotechnology is unique in several respects:

- It is both an Open Access journal and a printed journal.
- It is freely accessible worldwide – thanks to the Open Access approach.
- There are no publication fees for the authors and no costs for the readers.
- The copyright of the articles and figures is retained by the authors – they are allowed to use articles or the material contained in their articles freely, e. g., for inclusion on their website.
- Who pays? – The Beilstein Journal of Nanotechnology is completely funded by the Beilstein-Institut (http://www.beilstein-institut.de), a prestigious non-profit foundation for the advancement of science. The Beilstein-Institut has a long tradition and history in scientific publishing, and a high reputation, especially in the area of chemical sciences, dating back to 1951.
- There is no commercial interest behind the Beilstein Journal of Nanotechnology or the Beilstein-Institut. The aim of the journal is to support scientists in the field of nanoscience and nanotechnology and to provide the widest possible platform for cutting-edge research.
- All articles are peer-reviewed. The main criteria for acceptance of an article for publication are high quality, originality, novelty and importance.
- The members of the Editorial Board and the Advisory Board are leading scientists in the field of nanoscience and nanotechnology worldwide, including Jean-Marie Lehn and Sir Harold Kroto as Nobel laureates.

In this way, the Beilstein Journal of Nanotechnology, together with its sister journal, the Beilstein Journal of Organic Chemistry (http://www.bjoc.org), provides unique opportunities for the rapid and most effective dissemination of novel results. In addition to submitted articles, the concept of Thematic Series provides collections of invited, peer-reviewed contributions on selected focal areas of current interest, written by leading scientists in the specific field. If you are interested to initiate a Thematic Series, please contact the Editor-in-Chief.

It is my honour to thank all those who have contributed and who will contribute to the success of this journal. I would like to thank the authors of the articles – and our referees, renowned researchers in the field of nanotechnology, who ensure the scientific quality of the journal. Many thanks go to the members of the Advisory Board and the Editorial Board for their excellent support. Above all, and also on behalf of the authors, the Advisory Board and the Editorial Board, I would like to thank the Beilstein-Institut for launching and funding this unique journal.

It is my pleasure to invite you to become an active part in our journal and to profit from its advantages – as readers, as authors and as editors of Thematic Series.

Thomas Schimmel

Editor-in-Chief

Karlsruhe, November 2010

